# Strategies to develop clinical reasoning in nursing students: A structured review

**DOI:** 10.4102/hsag.v30i0.2889

**Published:** 2025-05-09

**Authors:** Adele Neethling, Lizeth Roets

**Affiliations:** 1 Department of Health Studies, College of Human Sciences, University of South Africa, Pretoria, South Africa

**Keywords:** clinical reasoning, learning, nursing education, teaching, teaching strategies

## Abstract

**Background:**

Clinical reasoning, a critical skill for nursing practitioners, has been linked to positive patient outcomes. While experienced nurses often demonstrate clinical reasoning abilities, evidence exist that it can be taught and mastered, making its development a key responsibility of nursing education institutions.

**Aim:**

To identify and describe teaching and learning strategies that can aid the development of clinical reasoning in nursing students.

**Method:**

A structured literature review was conducted using the following databases: EBSCO Host (Academic Search Ultimate, PsycINFO, MEDLINE, ERIC, and MasterFILE Premier), NEXUS (National Research Foundation), PubMed, and ProQuest Dissertations and Theses Full Text. Keywords included ‘nursing education’, ‘clinical reasoning’, ‘teaching’, ‘learning’, and ‘teaching strategies’ (*N* = 299). Filters reduced the number to 158, with a title review yielding 21 articles and the abstract review identified 18 articles for the final review.

**Results:**

Three main strategies were identified: (1)simulation, (2) classroom activities, and (3) e-learning. Classroom strategies yielded the most results (eight), followed by simulation (six) and e-learning (four). Each strategy incorporated various teaching modalities.

**Conclusion:**

All identified strategies and their modalities enhance clinical reasoning development.

**Contribution:**

Nursing faculties should consider incorporating these strategies in line with their teaching philosophy and available resources.

## Introduction

### Background

Nursing education institutions have a responsibility to enhance a positive practice environment; hence, the importance of the development of nursing students’ clinical reasoning skills is crucial for quality nursing care and improved patient outcomes (Neethling 2021). Even though this fact is known and well researched (Johnsen et al. 2016:39; Lee et al. 2016:20; Luo & Petrini 2018:175; Odajima & Furuichi 2020:399; Wuryanto et al. 2017:657), it is also found that nursing students are not well equipped with this important skill (Kim & KIm 2015:604; Dalton 2015:28; Koharchik et al. 2015:58). Even though there might be a play with words between different authors on what the definition of clinical reasoning is, it implies the process a nurse will follow to collect information from a patient, analyse the information, respond to the information collected and reflect on the effectiveness of the actions (Carvalho, Oliveira-Kumakura & Morais 2017). It is accepted that experienced or qualified nurses have better developed clinical reasoning skills than novice nurses; however, research findings have demonstrated that this skill, like most others in life, can be taught (Dumas, Torre & Durning 2018:709).

Previous reviews were primarily focused on specific teaching and learning approaches, such as simulation or e-learning, without recognising the value of other teaching and learning methods that also contribute valuable findings. Additionally, some reviews centred on the outcome – clinical reasoning – rather than exploring how students can be effectively taught.

This brought about the central question to be answered by the structured review: *Which teaching and learning strategies can be used to aid the development of clinical reasoning skills?*

## Methods

To answer the question, ‘which teaching and learning strategies can be used to aid the development of clinical reasoning skills?’, a structured review of the available literature was conducted. A structured review is an alternative form of a systematic review. This approach was utilised as even though the literature search was structured, it was not as rigorous as a full systematic review and a meta-analysis of the data was not performed. The advantage of a structured review over that of a systematic review is the use of a set of rules determined by the researcher, which could offer less bias and be more transparent in its execution of ensuring validity and reliability (Massaro, Dumay & Guthrie 2016).

### Search strategy

**FIGURE 1:**
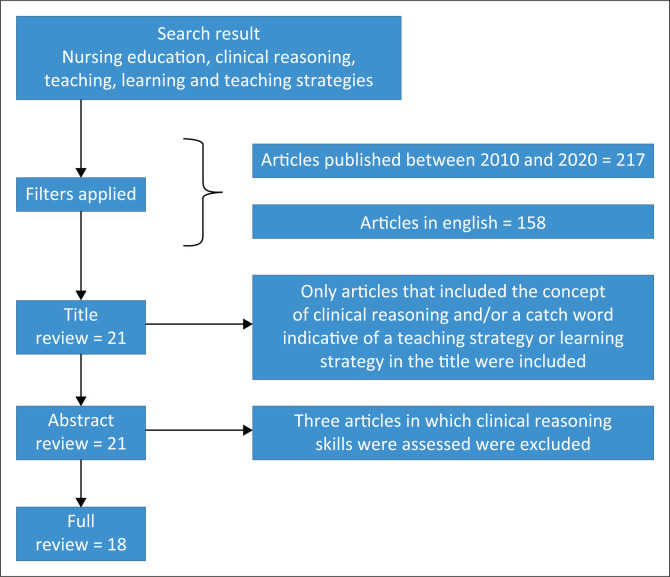
Search strategy.

A structured review of the literature was conducted with the aid of a specialist librarian allocated to the Department of Health Studies, responsible for supporting nursing education students and faculty with accessing relevant library resources and searches. The search engines used included EBSCOhost (Academic Search Ultimate, PsycINFO, MEDLINE, ERIC and MasterFILE Premier), NEXUS (National Research Foundation), PubMed, ProQuest Dissertations and Theses Full Text. The search terms used were ‘nursing education’, ‘clinical reasoning’, ‘teaching’, ‘learning’ and ‘teaching strategies’. The following filters were applied to limit the search results and identify more relevant articles: Only articles published between 2010 and 2020, full-text articles related to nursing education and those available in English were extracted. The collected articles were managed with Mendeley Reference Manager application and stored in folders and subfolders to aid the structured identification and elimination of articles through the review process. Figure 1 illustrates the implementation of the search strategy.

The search result yielded 299 results. A careful title review conducted by the authors, identifying articles, addressing clinical reasoning as a concept and indicating a specific teaching and learning strategy for the development thereof, was included in the selection (*n* = 21) (see Figure 1). The author and co-author independently reviewed all 21 abstracts and agreed that 18 articles complied with the inclusion criteria. Articles concerned with the assessment of clinical reasoning skills were excluded because of the irrelevance to the research question (*n* = 3). The selected 18 articles focused on the teaching and learning strategies aimed at developing clinical reasoning skills, underwent full review, and their relevance was assessed. Rigour was assured by involving a specialist librarian to assist with the literature search, as well as through the independent reviews of the authors. Both the librarian’s expertise and the independent reviews helped maintain a high standard of thoroughness and accuracy throughout the process.

### Quality analysis

The structured review aimed to determine which teaching and learning strategies were available to aid in developing clinical reasoning skills in nursing students. It focused on the teaching and learning strategies; therefore, relevant articles describing these teaching and learning strategies were considered (Table 1). This was done to ensure that a wide variety of teaching and learning strategies could be identified. Thus, it was not considered necessary to assess the quality of the articles included beyond ensuring that they matched the inclusion criteria.

**TABLE 1:** Relevance analysis.

Titles	Clinical reasoning in the title	Teaching and or learning strategy catch word in title	Design and technique	Ethical approval
A review of *clinical reasoning* in nursing education – based on *high-fidelity simulation* teaching method	Yes	Yes	Retrospective literature review	n/a
Effects of a *high-fidelity patient simulation-led clinical reasoning* course: Focused on nursing core competencies, problem-solving and academic self-efficacy	Yes	Yes	Quantitative, quasi-experimental study of non-equivalent control group pre-test–post-test design	Bioethics Committee of University College of Nursing (institutional review board no. 2012-0001)
Effects of *simulation* on nursing Students’ knowledge, *clinical reasoning* and self-confidence: A quasi-experimental study	Yes	Yes	Quantitative, quasi-experimental design with a control group	University institutional review boards (EU-12-02)
Enhancing *clinical reasoning* through *simulation debriefing*: a multisite study	Yes	Yes	Quantitative, quasi-experimental, pre-test–post-test, repeated measure	The study was approved by the institutional review board of each college/university
Using *clinical reasoning* and *simulation-based education* to ‘flip’ the enrolled nurse curriculum	Yes	Yes	Qualitative, model testing	n/a
Facilitating *clinical reasoning* in the *skills laboratory*: Reasoning model versus nursing process-based skills checklist	Yes	Yes	Quantitative, quasi-experimental	The university’s institutional review board exempted the study
Application of an *outcome present test-peer learning model* to improve *clinical reasoning* of nursing students in the intensive care unit	Yes	Yes	Qualitative Focus groups and interviews	Approved by: Faculty of Medical Research Ethical Committee – Universitas Gadjah Mada Institute of Research and Community Service (LPPM) of University of Muhammadiyah Semarang
*Clinical reasoning* in pre-licensure nursing students	Yes	No	Quantitative, quasi-experimental, one-group time-series	Permission obtained from the Institutional Review Board at Capella University and the dean of the nursing programme at the community college
Use of *outcome-present state test model of clinical reasoning* with Filipino nursing students	Yes	Yes	Quantitative, quasi-experimental comparison group pre-test/post-test	Loma Linda University (LLU) Institutional Review Board (IRB) Administrative Committee of the School
A *classroom activity* to enable nursing students to develop *clinical reasoning skills*	Yes	Yes	Quantitative ex post facto	Institutional Review Board of University of Phoenix and the community college
Development and validation of a *chronic disease nursing education programme* for enhancing *clinical reasoning* ability in undergraduate nursing students	Yes	Yes	Quantitative pre-test–post-test	Ethics committee of Nagoya City University Graduate School of Nursing, Nagoya, Japan
Fostering *clinical reasoning* in nursing students	Yes	No	Article	n/a
How does *questioning* influence nursing students’ *clinical reasoning* in problem-based learning – A scoping review	Yes	Yes	Scoping review	n/a
Use *of script-concordance activity* with the *think-aloud approach* to foster *clinical reasoning* in nursing students	Yes	Yes	Qualitative post-activity survey	n/a
Teaching *clinical reasoning* and decision-making skills to nursing students: Design, development and usability evaluation of a *serious game*	Yes	Yes	Qualitative deductive content analysis	Norwegian Social Science Data Service (no.38298)
*Technology-based strategies* for promoting *clinical reasoning skills* in nursing education	Yes	Yes	Literature review	n/a
The design and implementation of an *interactive computerised decision support framework (ICDSF)* as a strategy to improve nursing students’ *clinical reasoning skills*	Yes	Yes	Qualitative questionnaires	n/a
Theoretical foundations of educational strategies used in *e-learning* environments for developing *clinical reasoning* in nursing students: A scoping review	Yes	Yes	Scoping review	n/a

N/A, not applicable.

Of the 18 articles that were selected, 16 contained both the concept ‘clinical reasoning’ and a teaching and learning strategy ‘catch word’ in the titles. The two articles that did not contain a catch word indicative of a teaching strategy were included based on the abstract. Five of the articles were literature reviews, all of them related to teaching and learning strategies to aid the development of clinical reasoning skills or to support the development of clinical reasoning. Of the remaining 13 articles, eight were quantitative designs and five were qualitative designs. It was important to determine if the articles that included students as a vulnerable group of participants had ethical clearance. Ten of the 13 articles that used nursing students as part of their population had approval of some sort from their organisations. One of the articles related to a curriculum change, which assumed, was approved by the faculty. Students had to complete post-activity surveys, which only required students’ consent. Two of the articles related to teaching and learning strategies that were implemented in line with the curriculum, which also did not require permission. Both included surveys, which required students’ consent.

### Analysis

The authors read the selected articles to identify different types of teaching and learning strategies to develop clinical reasoning. The teaching and learning strategies were further refined into different teaching modalities. The articles’ titles were populated in Table 2, and a column for reviewing the effectiveness of each teaching modality in developing clinical reasoning skills was included. The analysis was reviewed by the co-author to ensure that content bias was excluded.

**TABLE 2:** Summary of results.

Titles	Author(s)	Teaching modalities	Recommendations
**Simulation**			
A review of clinical reasoning in nursing education – based on high-fidelity simulation teaching method	Luo, Q., Petrini, M.	High-fidelity simulation Number of contact sessions not defined†	Highly recommended Expensive Time consuming
Effects of a high-fidelity patient simulation-led clinical reasoning course: Focused on nursing core competencies, problem-solving and academic self-efficacy	Lee, J.H., Lee, Y., Lee, S., Bae, J.	High-fidelity simulation Clinical reasoning course Case scenario in simulation Debriefing †2 h/week for 16 weeks	Increase in core competency scores No impact on problem-solving and academic self-efficacy
Effects of simulation on nursing students’ knowledge, clinical reasoning and self-confidence: A quasi-experimental study	Kim, J.Y., Kim, E.J.	One-time simulation experience Attended theory class Completed case scenario in simulation Debriefing †2 scenarios	Simulation group scored significantly higher on clinical reasoning Must be incorporated in curriculum Expert knowledge to facilitate theory and practice during debriefing
Enhancing clinical reasoning through simulation debriefing: A multisite study	Forneris, S.G., Neal, D.O., Tiffany, J., Kuehn, M.B., Meyer, H.M., Blazovich, L.M., Holland, A.E., Smerillo, M.	Three unfolding case scenarios Each followed by debriefing for meaningful learning (DML). 1 simulation lab†	Nursing students who had DML debriefing scored significantly higher in their clinical reasoning than nursing students who had usual and customary debriefing Trained facilitators
Using clinical reasoning and simulation-based education to ‘flip’ the enrolled nurse curriculum	Dalton, L., Gee, T., Levett-Jones, T.,	Repository of videos, readings students work through independently Simulation-based practice session Discussion based on clinical reasoning cycle	Curriculum is designed to develop clinical reasoning
Facilitating clinical reasoning in the skills laboratory: Reasoning model versus nursing process-based skills checklist	Gonzol, K., Newby, C.	Identify, relate, understand, explain, predict, influence and control (IRUEPIC) in simulation Weekly sessions using medium fidelity for one semester†	The IRUEPIC reasoning model has the potential to assist clinical laboratory faculty in improving students’ ability to reason at a higher level, to apply theory during performance and to adapt to changing situations
**Classroom activities**			
Application of an outcome present test-peer learning model to improve clinical reasoning of nursing students in the intensive care unit	Wuryanto, E., Rahayu, G., Emilia, O., Octavia, A.P.	Outcome present state model – collaborative learning	Effective – facilitated guidance, triggered group process and strengthened self–directed learning
Clinical reasoning in pre-licensure nursing students	Harmon, M.M., Thompson, C.	Outcome present state model – collaborative learning †7 weeks	Improved clinical reasoning scores, still poor clinical reasoning
Use of outcome-present state test model of clinical reasoning with Filipino nursing students	Jael, S.A.	Outcome-present state test (OPT) model	The OPT Model of Clinical Reasoning, when used as a 2-week intervention programme, did not show significant improvement in nursing students’ clinical reasoning scores
A classroom activity to enable nursing students to develop clinical reasoning skills	Booher, C.D. (thesis)	Critical thinking teaching method – small groups	May be effective
Development and validation of a chronic disease nursing education programme for enhancing clinical reasoning ability in undergraduate nursing students	Odajima, Y., Furuichi, M.	Chronic disease nursing educational programme Lecture Case study – small group work presentation	Improved clinical reasoning scores
Fostering clinical reasoning in nursing students	Koharchik, L., Caputi, L., Robb, M., Culleiton, A.L.	Exercises – clinical conference: Tanner’s model – noticing, interpreting, responding, reflecting. Hypothetical case analysis using ISBAR. Reflection in-and-on action	Curriculum development aimed to foster clinical reasoning development
How does questioning influence nursing students’ clinical reasoning in problem-based learning – A scoping review	Merisier, S., Larue, C., Boyer, L.	Scoping review on questioning as a tool to enhance clinical reasoning	Not enough evidence to indicate questioning as a tool to improve clinical reasoning
Use of script-concordance activity with the think-aloud approach to foster clinical reasoning in nursing students	Tedesco-Schneck, M.	Script-concordance activity with the think-aloud approach (SCA-TA method) Answering questions Discuss the answers †6 classes	The SCA-TA method holds promise to foster CR. Ongoing research to predict the progression of CR is needed
**e-Learning**			
Teaching clinical reasoning and decision-making skills to nursing students: Design, development and usability evaluation of a serious game	Johnsen, H.M., Fossum, M., Vivekananda-Schmidt, P., Fruhling, A., Slettebø, Å.	Video-based serious games Video simulation Different levels of questioning	SG needs to have more feedback on right or wrong answers. Need correct software Need experts and technical support
Technology-based strategies for promoting clinical reasoning skills in nursing education	Shellenbarger, T., Robb, M.	Technology-based strategies such as electronic concept mapping, electronic case history and digital storytelling	Strategies that could be incorporated in curriculum
The design and implementation of an interactive computerised decision support framework (ICDSF) as a strategy to improve nursing students’ clinical reasoning skills	Hoffman, K., Dempsey, J., Levett-Jones, T., Noble, D., Hickey, N., Jeong, S., Hunter, S., Norton, C.	Interactive computerised decision support framework (ICDSF) †2 cases Case study Interaction Immediate feedback and remediation	They also believed that the ICDSF was useful in developing cognitive skills such as clinical reasoning, problem-solving and decision-making
Theoretical foundations of educational strategies used in e-learning environments for developing clinical reasoning in nursing students: A scoping review	Deschênes, M., Goudreau, J., Fontaine, G., Charette, M., Da Silva, K.B., Maheu-Cadotte, M., Boyer, L.	Scoping review – identify the types of educational strategies used in e-learning environments for developing or assessing CR in undergraduate nursing students. Tutorials Case studies, serious games and problem-based learning Animations Computerised interactive decision support	Strategies need a strong theoretical foundation

Note: Please see the full reference details of this article, https://doi.org/10.4102/hsag.v30i0.2889 for more information.

CR, clinical reasoning; SG, serious game.

† , the number of times/time duration students were exposed to a particular activity within the specific study.

### Ethical considerations

Ethical clearance to conduct this study was obtained from the University of South Africa Research ethics committee: Department of Health studies REC-012714-039 (NHREC), (reference no.: HSHDC/893/2019).

## Review findings

From the selected articles, three different teaching and learning strategies were identified as areas where clinical reasoning can be taught. The teaching and learning strategies included simulation, classroom activities as well as e-learning. The authors agreed that the complexities of patients’ conditions, the shorter stays in hospitals and the lack of appropriate support necessitated nursing education institutions to look for alternative ways to facilitate the development of clinical reasoning. Table 2 illustrates a summary of the results after the article analysis.

The results yielded six articles that highlighted simulation, eight articles that described classroom activities that can be incorporated and four articles that addressed e-learning as a teaching and learning strategy to develop clinical reasoning.

### Simulation as a teaching and learning strategy

Simulation teaching and learning strategies incorporated mostly high-fidelity simulation manikins with case scenarios that were followed by a debriefing session (Kim & KIm 2015:606; Lee et al. 2016:21; Luo & Petrini 2018:175). One article’s focus was on the importance and value of debriefing (Forneris et al. 2015:304), highlighting that simulation as an activity does not aid in clinical reasoning, but a structured debriefing is where clinical reasoning is developed. Dalton, Gee and Levett-Jones (2015:28–34) incorporated simulation as a strategy when they redesigned their nursing programme. Gonzol and Newby (2013:266) utilised simulation activities as a basis to determine which teaching methodology was more effective in developing clinical reasoning. The results from the articles regarding simulation as a teaching and learning strategy indicated that the nursing students involved showed improved clinical reasoning skills (Forneris et al. 2015:308; Gonzol & Newby 2013:266; Kim & KIm 2015:608). However, the cost involved to set up a high-fidelity simulation room and the time investment required to develop appropriate cases need to be carefully considered by the nursing faculty (Luo & Petrini 2018:181).

### Classroom activities as a teaching and learning strategy

The articles that described teaching activities that could be utilised in the classroom were very diverse. Use of the outcome-present state test (OPT) model for clinical reasoning development was described in three articles. In two of the articles, where the OPT was combined with collaborative teaching, the results showed improved clinical reasoning scores (Harmon & Thompson 2015:67; Wuryanto et al. 2017:659); however, one article found no change in the students’ clinical reasoning skills after the use of the OPT (Jael 2016:72). The remaining five articles described different classroom activities. These ranged from a once-off clinical conference (Koharchik et al. 2015:61), development of a clinical reasoning programme (Odajima & Furuichi 2020:400), a script concordance with a think-aloud approach (Tedesco-Schneck 2018:276) to a small group activity (Booher 2016:10). These four authors concluded that their activities improved clinical reasoning skills of the students. However, questioning as a tool to improve clinical reasoning was not proven effective (Merisier, Larue & Boyer 2018:114).

### e-Learning as a teaching and learning strategy

As a teaching and learning strategy, e-learning yielded the least number of articles in the search. Only four articles discussed technology-based modalities (excluding simulation) as teaching and learning strategies to enhance the development of clinical reasoning skills. Serious gaming, which entails case-based scenarios that encourage interaction from students (Hoffman et al. 2011:587–594; Johnsen et al. 2016:40), as well as traditional paper-based activities adapted to be used electronically (Deschênes et al. 2019:7; Shellenbarger & Robb 2015:80).

## Discussion

This article aimed to share the teaching and learning strategies that could aid in nursing students’ clinical reasoning development. Even though the identified teaching and learning strategies have been proven to improve clinical reasoning, the modality within these teaching and learning strategies needs careful consideration by the faculty. None of the identified modalities can be haphazardly implemented; they need to be planned for with the specific aim of developing clinical reasoning (Harmon & Thompson 2015:68; Koharchik et al. 2015:61; Merisier et al. 2018:114; Odajima & Furuich 2020:403; Tedesco-Schneck 2018:276; Wuryanto et al. 2017:659).

### Simulation modalities to enhance clinical reasoning

Simulation, whether high, medium or low fidelity, has been used for more than a decade to enhance nursing students’ clinical skills and theory application in a safe, controlled environment (Forneris et al. 2015:304; Dalton 2015:28–34; Kim & KIm 2015:604; Lee et al. 2016:21; Luo & Petrini 2018:175). The value of simulation on the development of clinical reasoning skills, however, lies in effective debriefing. The debriefing after a simulated scenario is where the students can reflect on their actions and explain the reasoning behind their decisions (Kim & KIm 2015:604; Lee et al. 2016:22). It is therefore important that the facilitator is skilled in debriefing and utilises an effective approach to structure the debriefing. Forneris et al. (2015:308) described the use of the debriefing for meaningful learning (DML) model to guide debriefing discussions as valuable to assist students to link their knowledge to their actions.

Luo & Petrini (2018:181) found that simulation has a positive impact on nursing students’ clinical reasoning skills, even though it is costly to implement. It is important to build simulation into the nursing curriculum and not just use it as a stand-alone teaching modality (Kim & KIm 2015:610; Luo & Petrini 2018:181). To use simulation effectively, the educators or those who are involved in education, both clinical and in theory, need to be skilled in creating meaningful scenarios and debriefing.

### Classroom modalities to enhance clinical reasoning

The OPT model has been highlighted by a number of authors as an effective tool to assist nursing students develop their clinical reasoning skills (Harmon & Thompson 2015:67; Wuryanto et al. 2017:661). The OPT uses a case study, whether a real patient history or a standardised case study, through which the students work. It consists of a reasoning web where students identify and group all the possible patient problems and in effect ‘order their thoughts’ to identify the patient’s main health issue. The students then worked through a frame plotting the patient’s current state and identifying what the ideal outcomes would be. This is followed by deciding on actions and tests that will allow the patient to progress from the current state to the desired outcome state. This approach was coupled with collaborative learning, which could be seen as an approach on its own (Harmon & Thompson 2015:65; Wuryanto et al. 2017:658). This classroom activity has been shown to have a remarkable impact on the development of students’ clinical reasoning skills (Wuryanto et al. 2017:662).

Other modalities, also referred to as activities, that were used in the classroom included the use of small groups of students completing tasks aimed at developing the clinical reasoning process. Booher (2016:64–65) used category cards for students to complete, based on a patient’s report. The category cards are circulated among the groups and then groups discuss their findings for each category and have an opportunity to explain their thought process. One other activity that made use of discussions to explain clinical reasoning process was described by Tedesco-Schneck (2018:276), where the script-concordance test was used as a basis for students to explain their thinking. This allowed the students to not only identify their mistakes but also reason as to why it was wrong or right.

Odajima and Furuichi (2020:401) implemented a programme consisting of five 90-min sessions, which included a lecture, group work and presentations. The programme was based on the nursing process, with each session guiding the student through the steps of the nursing process, helping them to develop a deeper understanding of required nursing care to be given to a specific patient. Koharchik et al. (2015:59) described the implementation of Tanner’s clinical reasoning model while engaging with students, using questioning techniques that allow students to think about the presented information and reason through the process. However, (Merisier et al. 2018:108–115) found that questioning is a technique used by educators and facilitators to encourage students to think rather than teaching them to reason. Koharchik et al. (2015:60) further described how the ISBAR (identification-situation-background-assessment-recommendation) tool with a hypothetical case can aid students to develop their reasoning.

### e-Learning modalities to enhance clinical reasoning

e-Learning is a teaching and learning strategy that is being used increasingly in nursing education (Deschênes et al. 2019:1; Shellenbarger & Robb 2015:79). Some modalities used include electronic concept mapping, electronic case presentations and serious gaming, among others. Shellenbarger and Robb (2015:80) found that electronic concept mapping enhances the learning experience, as amendments or additions using concept mapping software are easier than adapting a paper and pen concept map. The use of an electronic case presentation is also aimed at developing clinical reasoning environment (Shellenbarger & Robb 2015:80). Creating a case scenario with hyperlinks to additional reading, YouTube videos or other related case material was aimed at engaging students and aligning the activity as close to reality as possible.

Serious gaming is an e-learning approach derived from simulation as a teaching modality. The students are engaged in a virtual scenario simulating a real environment (Johnsen et al. 2016:40). This gaming software has been found to engage students, and through careful questioning, can aid in the development of clinical reasoning (Johnsen et al. 2016:45).

It is, however, noticeable that all the teaching and learning strategies and inclusive modalities were based in a nursing institution, either a classroom, simulation room or computer laboratory. None of the teaching and learning strategies included nursing practice and interaction with actual patients to develop clinical reasoning skills. As clinical reasoning develops more with the experience a nurse gains (Koharchik et al. 2015:59), it is understood that planning for opportunities where nursing students can implement their clinical reasoning skills they are taught in the nursing college or institution is key to nursing practice. It is therefore important to determine if the results seen with the research done can be transferred to the students’ use of clinical reasoning while busy with their clinical programme. Neethling (2021) applied a cooperative clinical reasoning activity, which had to be completed by the students using a real patient’s health information to develop their clinical reasoning skills. This cooperative learning activity showed that the students developed clinical reasoning skills significantly more than those who did not participate in the activity.

### Limitations

The authors acknowledge that the strict search criteria, which limited results to articles with the phrases ‘teaching strategies’ or ‘learning strategies’ in the title, may have resulted in the exclusion of relevant articles.

## Conclusion

After the review, it was evident that any one of the identified teaching and learning strategies and supporting modalities could aid in developing clinical reasoning skills. It would be necessary for the nursing faculty to choose and align the choice of strategy to the teaching philosophy to determine the best teaching and learning strategy(s) to apply for clinical reasoning development. Simulation and e-learning as teaching strategies can be very costly (Luo & Petrini 2018:181) to implement initially and involve continued training of faculty, technical support and time for development (Shellenbarger & Robb 2015:81). If e-learning and simulation do not have proper technical support, students might feel they are wasting their time and missing out on an effective learning opportunity (Hoffman et al. 2011:593).

With these modalities (simulation and e-learning), it was clear that there were planned facilitator interaction throughout, which guided the students to achieve the development of their clinical reasoning skills (Booher 2016:64–66; Harmon & Thompson 2015:68; Koharchik et al. 2015:61; Odajima & Furuichi 2020:401; Tedesco-Schneck 2018:276; Wuryanto et al. 2017:659). A commonality among the teaching and learning strategies was the use of collaborative learning in some of the modalities. Collaborative learning has been proven to enhance clinical reasoning skills development in nursing students (Tedesco-Schneck 2018:277). Even though none of the classroom activities focused on collaborative learning as an approach, and rather used it as a vehicle to deliver the activities, the inclusion of collaborative learning as an approach could be the key to the success of the modalities. It is also clear that there is not one teaching and strategy that is considered the best strategy. As mentioned before, it either will be up to the nursing faculty to decide on any of the proven teaching and learning strategies, individually or combined, to include in the nursing curriculum.
